# Immune Checkpoint-Associated Locations of Diffuse Gliomas Comparing Pediatric With Adult Patients Based on Voxel-Wise Analysis

**DOI:** 10.3389/fimmu.2021.582594

**Published:** 2021-03-17

**Authors:** Li Zhang, Buyi Zhang, Zhangqi Dou, Jiawei Wu, Yasaman Iranmanesh, Biao Jiang, Chongran Sun, Jianmin Zhang

**Affiliations:** ^1^Department of Oncology, Daqing Oilfield General Hospital, Daqing, China; ^2^Department of Pathology, School of Medicine, The Second Affiliated Hospital of Zhejiang University, Hangzhou, China; ^3^Department of Neurosurgery, School of Medicine, The Second Affiliated Hospital of Zhejiang University, Hangzhou, China; ^4^Department of Radiology, School of Medicine, The Second Affiliated Hospital of Zhejiang University, Hangzhou, China

**Keywords:** pediatric diffuse gliomas, immune checkpoint molecules, spatial locations, B7-H3, CD47, PD-L1, immunotherapy

## Abstract

**Objective:** Pediatric diffuse gliomas (pDGs) are relatively rare and molecularly distinct from pediatric pilocytic astrocytoma and adult DGs. Immunotherapy is a promising therapeutic strategy, requiring a deep understanding of tumor immune profiles. The spatial locations of brain tumors might be related to the molecular profiles. We aimed to analyze the relationship between the immune checkpoint molecules with the locations of DGs comparing pediatric with adult patients.

**Method:** We studied 20 pDGs patients (age ≤ 21 years old), and 20 paired adult patients according to gender and histological types selected from 641 adult patients with DGs. Immune checkpoint molecules including B7-H3, CD47, and PD-L1, as well as tumor-infiltrating lymphocytes (TILs) and tumor-associated macrophages (TAMs), were manifested by immunohistochemical staining. Expression difference analyses and Spearman's correlation were performed. MRI data were voxel-wise normalized, segmented, and analyzed by Fisher's exact test to construct the tumor frequency and p value heatmaps. Survival analyses were conducted by Log-rank tests.

**Result:** The median age of pediatric patients was 16 years. 55% and 30% of patients were WHO II and III grades, respectively. The left frontal lobe and right cerebellum were the statistically significant locations for pDGs, while the anterior horn of ventricles for adult DGs. A potential association between the expression of PD-L1 and TAMs was found in pDGs (p = 0.002, R = 0.670). The right posterior external capsule and the lateral side of the anterior horn of the left ventricle were predominant locations for the adult patients with high expression of B7-H3 and low expression of PD-L1 compared to pediatric ones, respectively. Pediatric patients showed significantly improved overall survival compared with adults. The prognostic roles of immune checkpoint molecules and TILs/TAMs were not significantly different between the two groups.

**Conclusion:** Immune checkpoint-associated locations of diffuse gliomas comparing pediatric with adult patients could be helpful for the immunotherapy decisions and design of clinical trials.

## Introduction

The incidence of CNS tumors in children and adolescents in the United States is 6.06 per 100,000 according to the latest CBTRUS statistical report (1). High childhood cancer-related mortality is observed in pediatric patients with CNS tumors, which is the second most malignancy after leukemia (2). Pediatric gliomas are the most common type therein, and the low-grade gliomas (WHO grades I and II) constitute a majority of pediatric gliomas, such as pilocytic astrocytoma and subependymal giant cell astrocytoma (3). The high-grade gliomas (WHO grades III and IV) are relatively rare but extremely fatal (4). Pediatric diffuse gliomas (pDGs) are a subgroup of pediatric gliomas that histologically including anaplastic/non-anaplastic astrocytoma, oligodendroglioma, oligoastrocytoma, and glioblastoma multiform (5). Patients with pDGs are highly heterogeneous and differ from the adult counterparts and the common pediatric gliomas such as pilocytic astrocytoma molecularly, clinically, and prognostically (4, 6, 7). However, the distinctive characteristics of pDGs remain largely unknown.

Neuroimaging including CT and MRI is a pivotal method to detect the pDGs. Imaging information include tumor location, volume, and edema which are associated with clinical symptoms. It was reported that less neurologic impairments of pediatric gliomas were observed when tumors located at cerebral hemispheres compared with ones at midline, optic pathway, posterior fossa, and brainstem (8). Besides the advantages in the determination of symptom and diagnosis, the tumor location in radiology promotes the accuracy of surgical resection and the efficacy of outcome evaluation (9–11).

Immunotherapy is an emerging approach treating the refractory gliomas in addition to surgery and chemoradiotherapy. Since the chemoradiotherapy may cause developmental disorders and other side effects in pediatric patients, immunotherapy becomes the novel alternative for glioma management (12). Checkpoint inhibitors work by promoting the antitumor immune response. The effect of PD-1 blockage Nivolumab was investigated in patients with recurrent glioblastoma but the overall survival was not improved, probably due to the low permeability of blood-brain barrier and suppressive immune microenvironment (13). Notably, immunotherapy is facing more challenges in pediatric gliomas, a unique group differing from adult patients (14). For instance, the expression of immune checkpoint molecules may be highly distinct between pediatric and adult gliomas.

In the present study, using voxel-wise analysis, we aim to investigate the association between the spatial locations of pDGs and the expression of immune checkpoint molecules including B7-H3, CD47, and PD-L1, as well as the tumor-infiltrating lymphocytes (TILs) and tumor-associated macrophages (TAMs), when compared to the adult DGs. The location-associated immune characteristics may be valuable for the design of the immunotherapy regimen.

## Materials and Methods

### Patient Cohort

Patients diagnosed with brain tumors from March 2012 to December 2017 were initially searched in our institutional database and 2,048 patients were reviewed. The histopathological data and preoperative craniocerebral contrast-enhanced MRI were collected. A total of 661 patients with DGs including anaplastic/non-anaplastic astrocytoma, oligodendroglioma, oligoastrocytoma, and glioblastoma multiform were confirmed. Pediatric (aged ≤ 21 years) patients were grouped into one cohort, adults into the other (15). Among the adult patients, 20 were randomly selected and paired according to the gender and histological types of every pediatric patient for further analyses.

### Patient Consent

This study was approved by the ethical committee on clinical human research in the institution (No. 2020-876). The informed consent of the pathological examination of surgical specimens was signed by every patient as soon as the admission to hospital. Because of no clinical intervention, the committee had waived the specific informed consent agreement for the review of clinical information and imaging data in the current study.

### Magnetic Resonance Imaging

The contrast-enhanced MRI was acquired in patients with intravenous injection of gadodiamide (0.2 ml/kg body weight, up to a maximum of 20 ml, Omniscan, GE Healthcare) followed by the use of 1.5 (Signa Excite, GE Healthcare, Milwaukee, Wisconsin) or 3.0 Tesla (Discovery 750, GE Healthcare, Milwaukee, Wisconsin) MRI.

### Imaging Data

The imaging data in the standard Digital Imaging and Communications in Medicine (DICOM) format were converted to the Neuroimaging Informatics Technology Initiative (NIfTI) format using dcm2nii converter software (University of Nottingham School of Psychology, Nottingham, UK). The data were registered to the standard brain template (MNI152; Montreal Neurological Institute, McGill University, Montreal, Quebec, Canada) using Statistical Parametric Mapping Software version 12 (SPM12, Institute of Neurology, University College London, London, UK) in MATLAB (version R2012a, The MathWorks, Natick, MA, USA). The regions of interest (ROIs) were obtained after the semiautomatic segmentation of normalized data using 3D Slicer (version 4.10.0; http://www.slicer.org/) (16). The processes were conducted by trained authors (LZ and ZD), and were reviewed by two experienced neurosurgeons and a neuroradiologist (CS, JZ, and BJ).

### Construction of Frequency and *P*-Value Heatmaps

To visualize the spatial distribution of DGs, the ROIs were overlapped on the MNI152 by MRIcron (University of Nottingham School of Psychology, Nottingham, UK) to create frequency heatmaps. The *p*-value heatmaps comparing two different phenotypes (e.g., pediatric patients and adult ones) were constructed using the analysis of differential involvement (ADIFFI) as previously described by Ellingson et al. (17, 18). Briefly, a 2×2 contingency table was used to perform a two-tailed Fisher's exact test for the significance calculation of a particular voxel:

$$\begin{array}{l} p=\frac{\left(a+b\right)!\left(c+d\right)!\left(a+c\right)!\left(b+d\right)!}{a!b!c!d!n} \end{array}$$

In the formula, “a” is the frequency of tumor occurrence under phenotype A, “b” is the frequency of tumor occurrence under phenotype B, “c” is the frequency of tumor-free patients under phenotype A, “d” is the frequency of tumor-free patients under phenotype B, and “n” is the total number of patients. The exclamation mark refers to the factorial operation.

### Immunohistochemical Staining

The DGs tissues were fixed, dehydrated, and paraffin-embedded. The 4 μm sections were deparaffinized and rehydrated in 100, 95, and 75% ethanol. The antigen retrieval solution with EDTA (pH 9.0) was used for PD-L1 staining, while the solution with sodium citrate (pH 6.0) for B7-H3 and CD47 staining. After endogenous peroxidase activity blocking, the sections were rinsed and incubated with the primary antibodies including anti-human PD-L1 (1:1,000, Abcam, ab228462, Cambridge, MA, USA), CD47 (1:2000, Abcam, ab218810) and B7-H3 (1:2,000, Abcam, ab219648) overnight at 4°C. TILs were stained by the CD45 (1:1,000, Abcam, ab40763) antibody, and TAMs were stained by the CD68 antibody (1:1,000, Abcam, ab213363). Images were acquired after the incubation with secondary antibodies and DAB, and counterstained with Hematoxylin. The expression level of three immune checkpoint molecules was determined by the percentage of positive cells and the staining intensity: low (negative intensity and intensity 1, and intensity 2 with positive cells < 10%) and high (intensity 2 with positive cells ≥ 10% and intensity 3) expression (19). The expression of TILs/TAMs was similarly evaluated as previously described (20, 21).

### Statistical Analysis

The data were presented as the mean ± standard error of mean (SEM) by nonparametric paired *t*-test. The paired four-fold table was statistically analyzed by McNemar's test. Spearman's correlation analysis was performed between immune checkpoints and TILs/TAMs. The two-tailed Fisher's exact test was mentioned above. Overall survival (OS) was defined as the time of imaging detection until death or the last follow-up. The Kaplan-Meier analysis with the Log-rank test was conducted to evaluate the OS. We used GraphPad Prism (version 8.0.2; GraphPad Software, San Diego, CA, USA) and SPSS (version 22.0; IBM SPSS Statistics, Armonk, NY, USA) for all statistical analyses. *P* < 0.05 was considered significant.

## Results

### Demographics

A total of 20 pediatric patients (age ≤ 21 years old) with DGs were analyzed. The demographics including age, gender and histological types were listed in Table 1. The median age of pediatric patients was 16 years. There were 55% of WHO grade II DGs. According to the gender and histological types of pediatric patients, 20 adult patients (age > 21 years old) from the adult cohort (641 patients) were randomly paired. In the entire cohort (661 patients), the percentage of patients with glioblastoma was 53.9% (Table 1).

**Table 1:** Demographics of all patients with diffuse gliomas and 20 pediatric patients with diffuse gliomas, as well as 20 paired adult patients based on the gender and histological types of pediatric patients.

**Characteristics**	**All patients with diffuse gliomas** **(*n* = 661)**	**Pediatric patients with diffuse gliomas** **(*n* = 20)**	**Paired adult patients with diffuse gliomas** **(*n* = 20)**
**Age (years)**			
Range	6–87	6–21	27–68
Median	51	16	43
**Gender (%)**			
Male	385 (58.2)	16 (80)	16 (80)
Female	276 (41.8)	4 (20)	4 (20)
**Histological type (%)**			
Diffuse astrocytoma	57 (8.6)	4 (20)	4 (20)
Oligodendroglioma	32 (4.8)	5 (25)	5 (25)
Oligoastrocytoma	22 (3.3)	2 (10)	2 (10)
Anaplastic astrocytoma	89 (13.5)	3 (15)	3 (15)
Anaplastic oligodendroglioma	58 (8.8)	1 (5)	1 (5)
Anaplastic oligoastrocytoma	47 (7.1)	2 (10)	2 (10)
Glioblastoma	356 (53.9)	3 (15)	3 (15)

### Left Frontal Lobe and Right Cerebellum Were the Preferred Locations for pDGs

**Figure 1:**
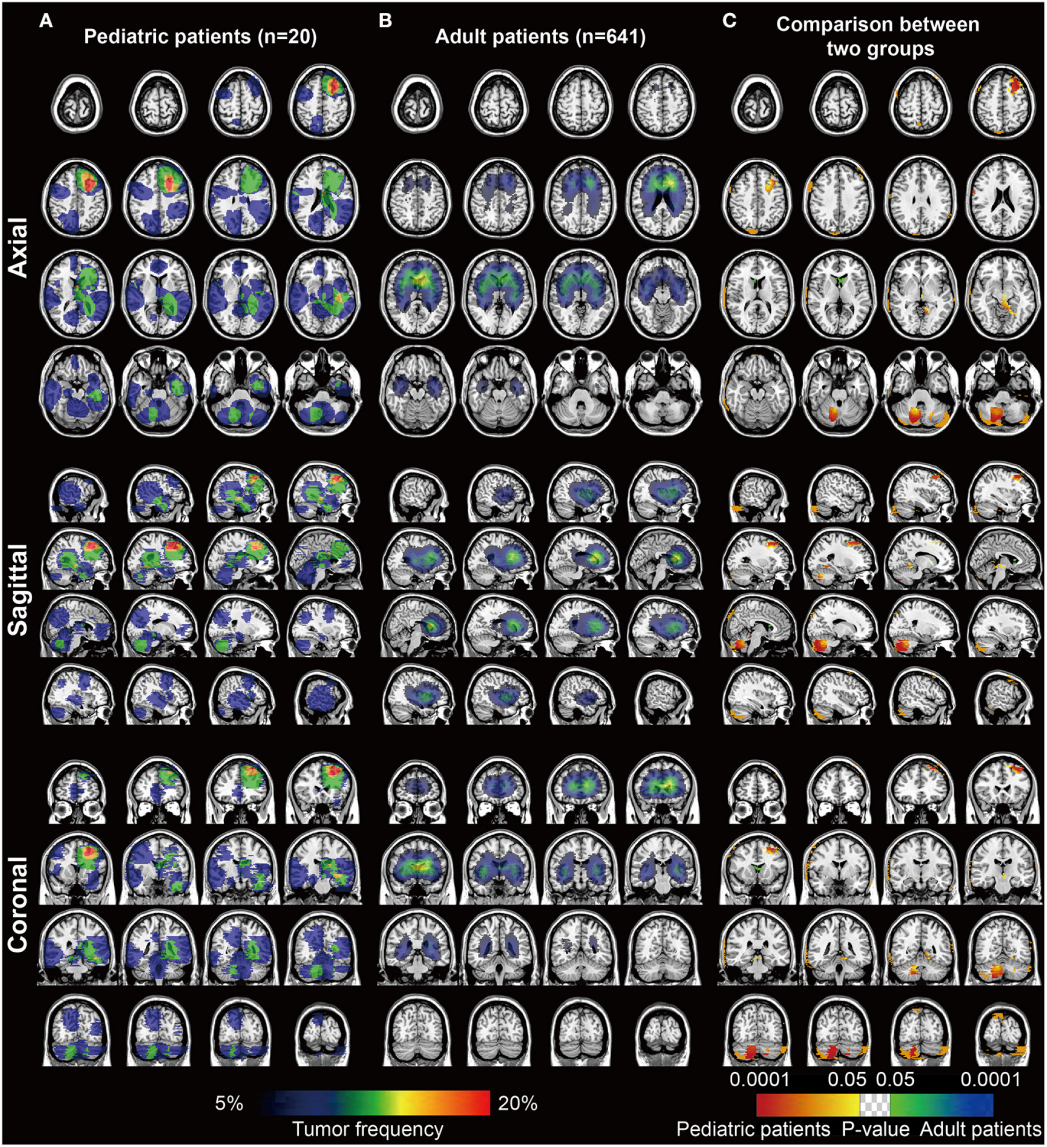
The frequency and p value heatmaps in axial, sagittal, and coronal positions comparing pediatric and adult patients with DGs. **(A)** The frequency heatmap of pDGs. **(B)** The frequency heatmap of adult DGs. The color ranging from dark blue to red suggested the tumor frequency from 5 to 20% and above. **(C)** The p value heatmap comparing the two groups after the Fisher's exact test. The color ranging from dark blue to green, and red to bright yellow, both suggested the *p*-value from 0.0001 to 0.05.

The frequency heatmaps of DGs were constructed by ROIs overlapping to display the spatial distribution of DGs. The color ranging from dark blue to red suggested the tumor frequency from 5 to 20% and above. For pediatric patients, left frontal lobe showed relatively high incidence of pDGs (Figure 1A). For the remaining 641 patients with DGs, the paraventricular and subventricular regions, especially the anterior horn of lateral ventricles, were frequently affected (Figure 1B). By using Fisher's exact test, significant clusters for pDGs were identified in the left frontal lobe and right cerebellum, and clusters for adult DGs in the anterior horn of ventricles (*p* < 0.05, Figure 1C).

### IHC Results of B7-H3, CD47, PD-L1 and TILs/TAMs

**Figure 2:**
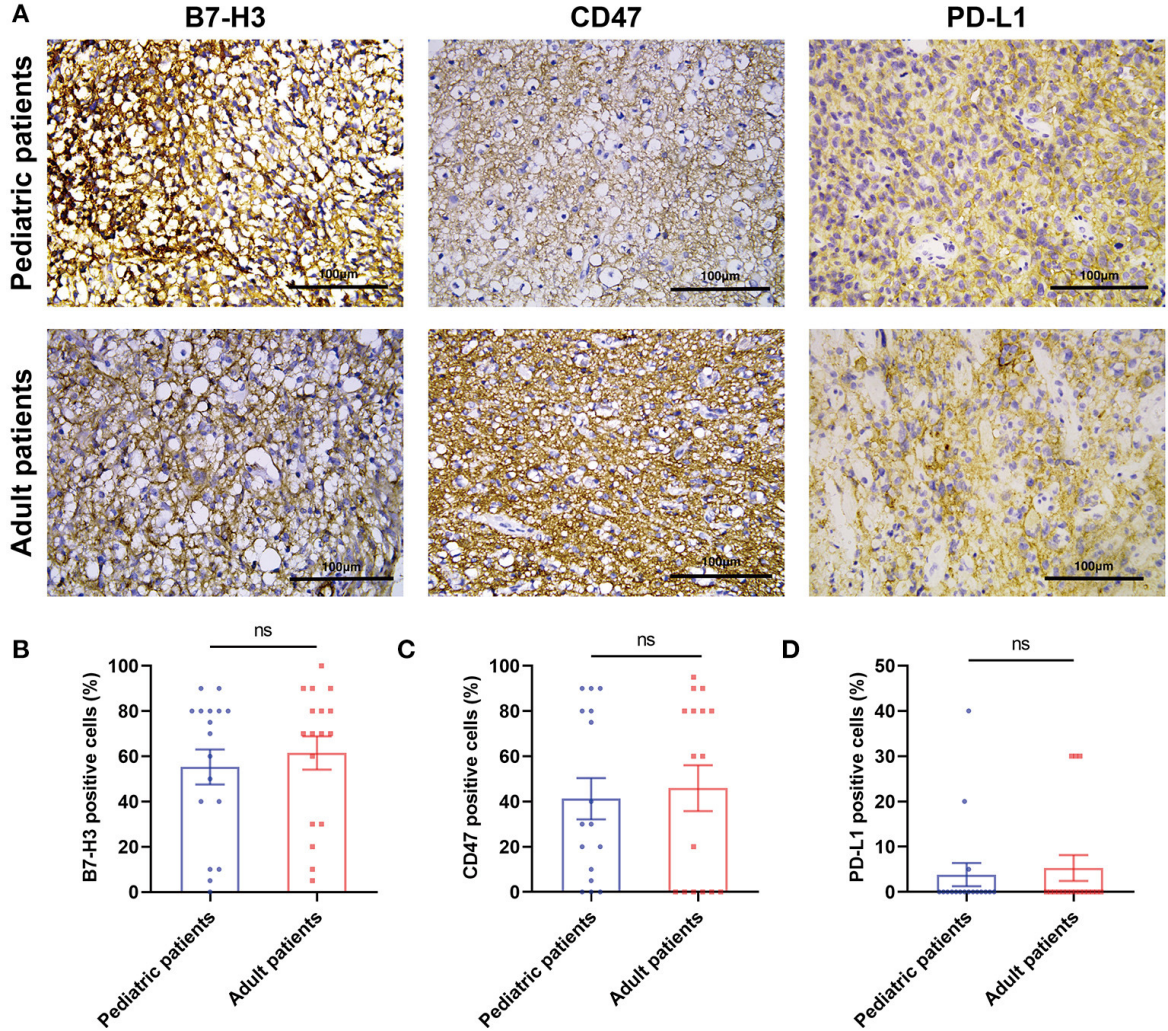
The IHC results of three immune checkpoint molecules in pediatric and adult patients with DGs. **(A)** Representative images of IHC staining of B7-H3, CD47, and PD-L1 in pediatric and paired adult patients with DGs. The scale bar is 100 μm. **(B)** Paired *t*-test analyzing the percentage of B7-H3 positive cells in the two groups. **(C)** Paired *t*-test analyzing the percentage of CD47 positive cells in the two groups. **(D)** Paired *t*-test analyzing the percentage of PD-L1 positive cells in the two groups. The ns refers to “not significant”.

Three immune checkpoints molecules, B7-H3, CD47 and PD-L1 were immunohistochemically stained in pediatric and paired adult DGs, as shown in Figure 2A. To reveal the difference of checkpoints expression between pediatric and paired adult DGs, the positive cells, and patient quantity with high or low expression (determined by positive cells and staining intensity), were respectively compared. However, no significance was found (Table 2 and Figures 2B–D). Furthermore, the expression level of TILs (CD45 staining) and TAMs (CD68 staining) were determined by IHC, and no significance was either found comparing patient quantity (Table 2).

**Table 2:** Patient quantity in terms of three immune checkpoints, TILs and TAMs.

	Pediatric patients^*^		Paired adult patients^*^		***P*-value^#^**
	**Patient quantity with high expression**	**Patient quantity with low expression**	**Patient quantity with high expression**	**Patient quantity with low expression**	
B7-H3	12	7	12	6	0.359
CD47	7	12	6	12	0.238
PD-L1	1	18	1	17	NA
TILs	7	12	8	10	0.503
TAMs	7	12	4	14	0.077

* *Because of the loss of specimens, the total number was not 20*.

# *The p-value was calculated by McNemar's test*.

*TILs refer to the tumor infiltrating-lymphocytes, and TAMs refer to the tumor-associated macrophages. NA refers to “not available”*.

The relationship between immune checkpoints molecules and immune cells such as lymphocytes and macrophages was profoundly studied. Therefore, the Spearman's correlation was performed to analyze the potential relationship in the current study (Table 3). For pediatric patients, the percentage of positive cells of PD-L1 was significantly correlated to the expression level of TAMs (*p* = 0.002, *R* = 0.670). For the group of pediatric and adult patients, the expression level of TAMs was significantly correlated to the B7-H3 (*p* = 0.009, *R* = 0.428) and PD-L1 (*p* = 0.005, *R* = 0.458). No significant correlation was found in the paired adult group. The results may indicate the distinct role of macrophages in pDGs.

**Table 3:** Spearman's correlation of TILs/TAMs with the immune checkpoints.

**Spearman's correlation**		**TILs**			**TAMs**		
		**Pediatric patients**	**Paired adult patients**	**Pediatric and paired adult patients**	**Pediatric patients**	**Paired adult patients**	**Pediatric and paired adult patients**
B7-H3	*P*-value	0.537	0.053	0.080	0.087	0.226	**0.009**
	*R*	0.151	0.477	0.296	0.403	0.300	**0.428**
CD47	*P*-value	0.651	0.439	0.420	0.298	0.086	0.070
	*R*	−0.111	−0.201	−0.139	−0.252	−0.429	−0.306
PD-L1	*P*-value	0.569	0.426	0.308	**0.002**	0.637	**0.005**
	*R*	0.140	0.200	0.175	**0.670**	0.120	**0.458**

*P-values with statistical significance and the corresponding R are boldface*.

*TILs refer to the tumor-infiltrating lymphocytes, and TAMs refer to the tumor-associated macrophages*.

### Immune Checkpoint-Associated Locations of DGs

**Figure 3:**
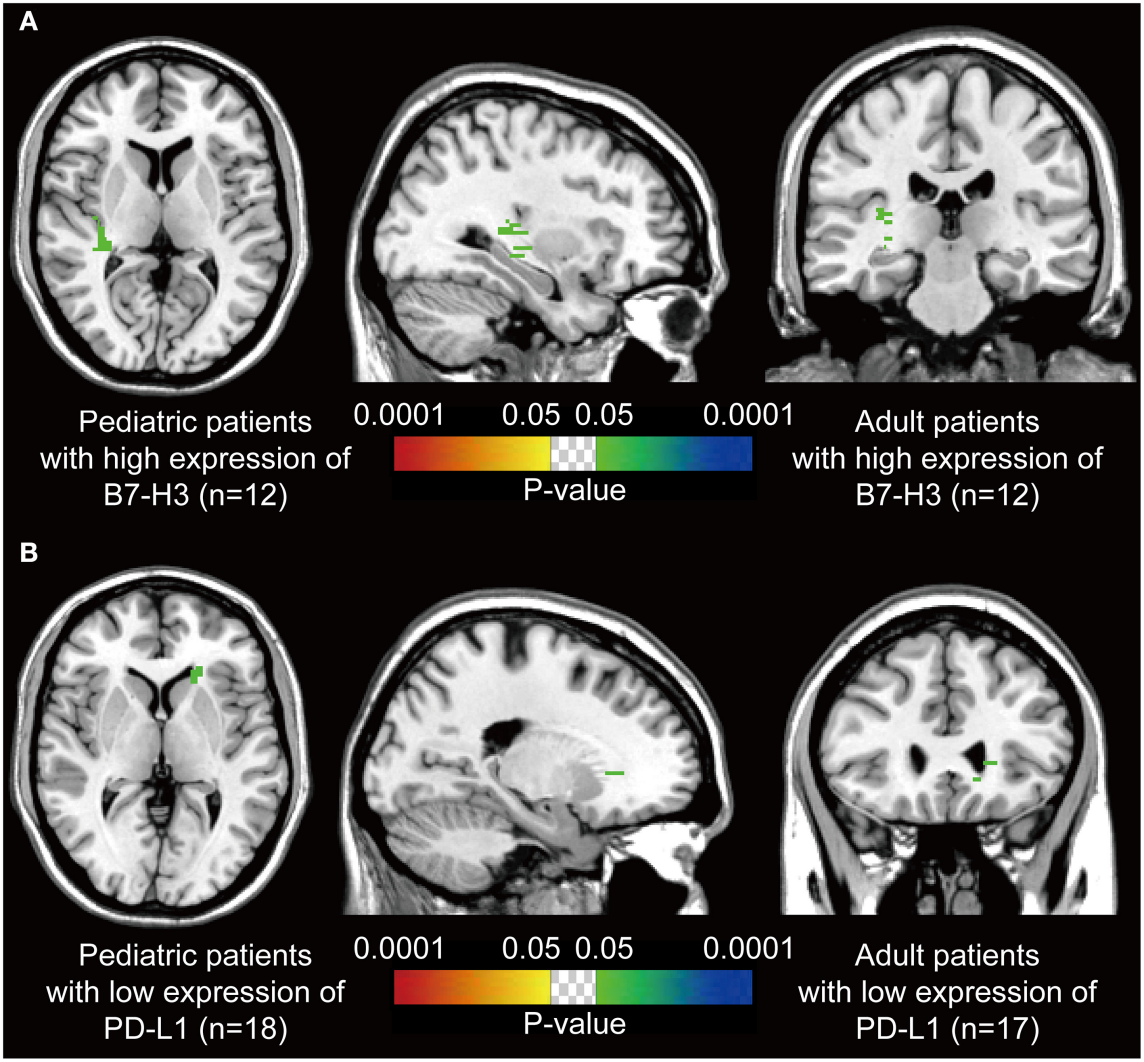
*P*-value heatmaps visualize the immune checkpoint-associated locations of DGs. **(A)** The *p*-value heatmap showed the right posterior external capsule was the predominant location for the adult DGs with high expression of B7-H3 compared to pediatric ones. **(B)** The *p*-value heatmap showed the lateral side of the anterior horn of the left ventricle was the predominant location for the adult DGs with low expression of PD-L1 compared to pediatric ones. The color ranging from dark blue to green, and red to bright yellow, both suggested the *p*-value from 0.0001 to 0.05.

We assumed that the location of DGs may be associated with the immune characteristics. Thus, voxel-wise Fisher's exact test was applied to visualize the significant clusters by comparing the pediatric and the paired adult groups based on the expression level of immune checkpoints. Paired adult patients with high expression of B7-H3 displayed a remarkable location in the right posterior external capsule compared to the pediatric group (*p* < 0.05, Figure 3A). The lateral side of the anterior horn of the left ventricle was identified as a distinct location in paired adult patients with low expression of PD-L1 compared to the pediatric group (*p* < 0.05, Figure 3B). The checkpoint-associated locations of DGs may provide the potential value for immune therapeutic strategies according to the location of the tumor.

### Survival Differences According to the Expression Level of Checkpoints and TILs/TAMS

**Figure 4:**
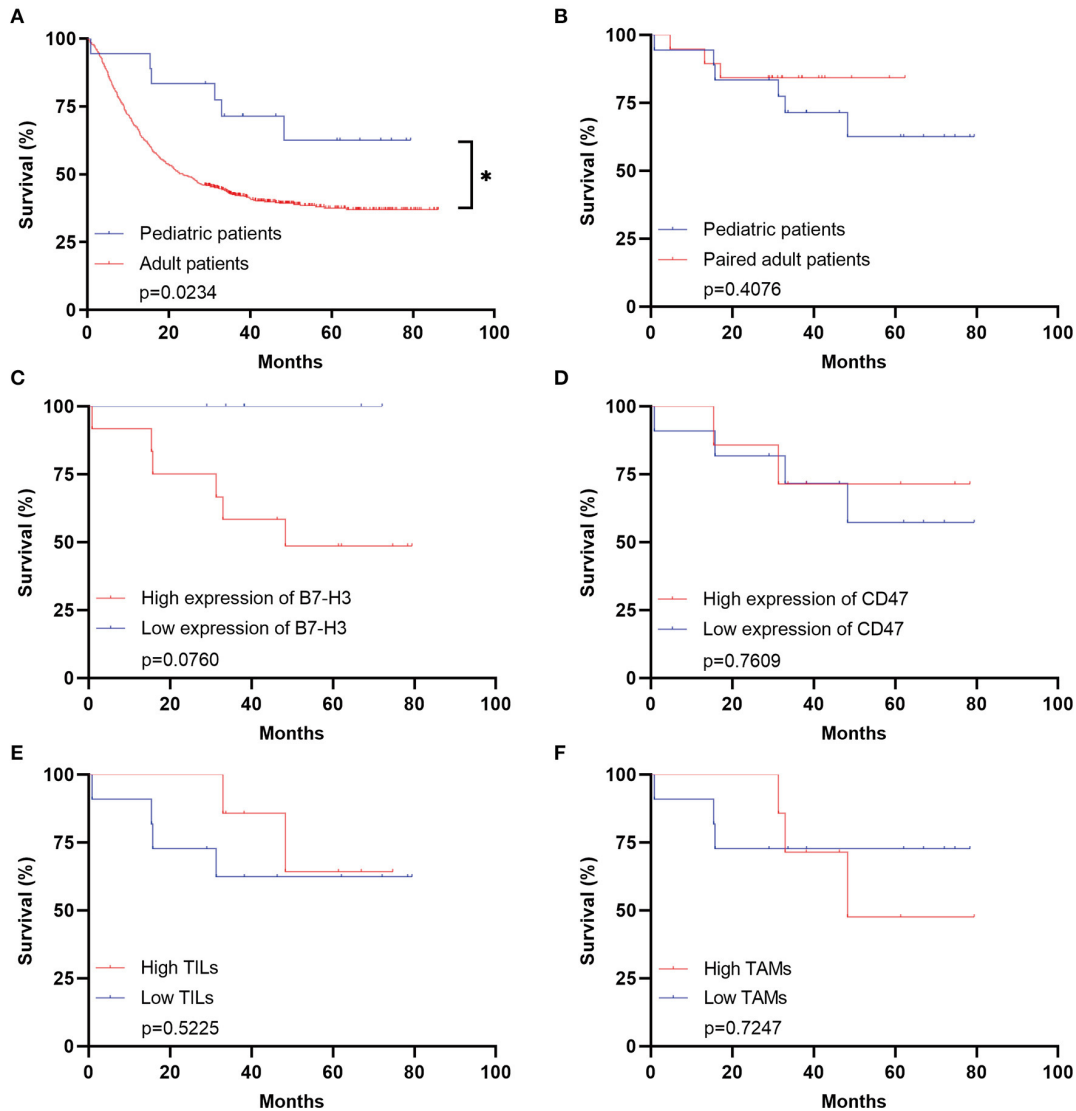
Survival analyses for pediatric patients with DGs. **(A)** Survival analysis comparing the pediatric group with the adult group (*n* = 641, *p* = 0.0234). **(B)** Survival analysis comparing the pediatric group with the paired adult group (*n* = 20, *p* = 0.4076). **(C)** Survival analysis comparing the high expression of B7-H3 with the low expression in the pediatric group (*p* = 0.0760). **(D)** Survival analysis comparing the high expression of CD47 with the low expression in the pediatric group (*p* = 0.7609). **(E)** Survival analysis comparing the high TILs with the low TILs in the pediatric group (*p* = 0.5225). **(F)** Survival analysis comparing the high TAMs with the low TAMs in the pediatric group (*p* = 0.7247). TILs refer to the tumor-infiltrating lymphocytes; TAMs refer to the tumor-associated macrophages; * refers to the *p* < 0.05.

**Figure 5:**
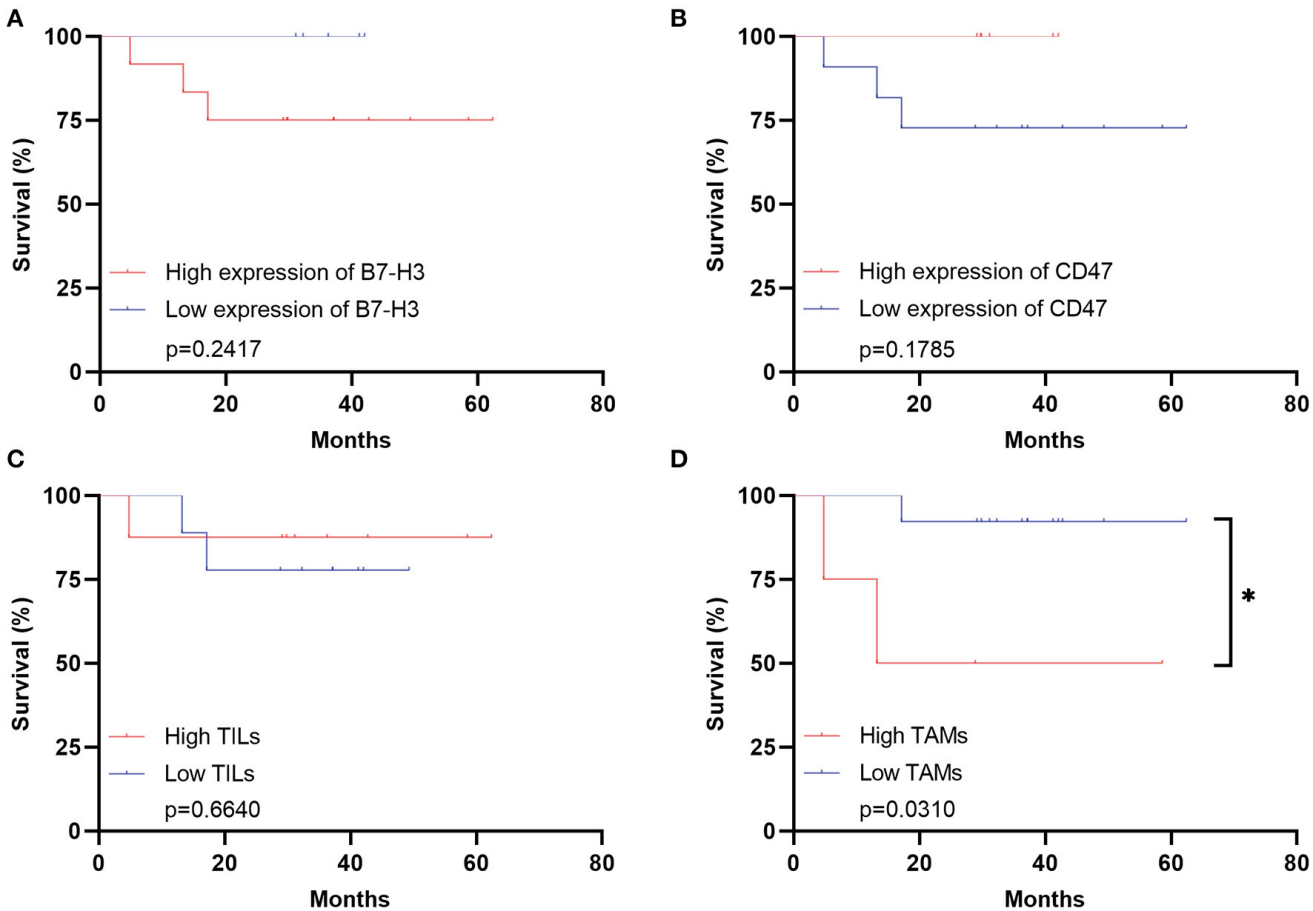
Survival analyses for adult patients with DGs. **(A)** Survival analysis comparing the high expression of B7-H3 with the low expression in the adult group (*p* = 0.2417). **(B)** Survival analysis comparing the high expression of CD47 with the low expression in the adult group (*p* = 0.1785). **(C)** Survival analysis comparing the high TILs with the low TILs in the adult group (*p* = 0.6640). **(D)** Survival analysis comparing the high TAMs with the low TAMs in the adult group (*p* = 0.0310). TILs refer to the tumor-infiltrating lymphocytes; TAMs refer to the tumor-associated macrophages; * refers to the *p* < 0.05.

**Figure 6:**
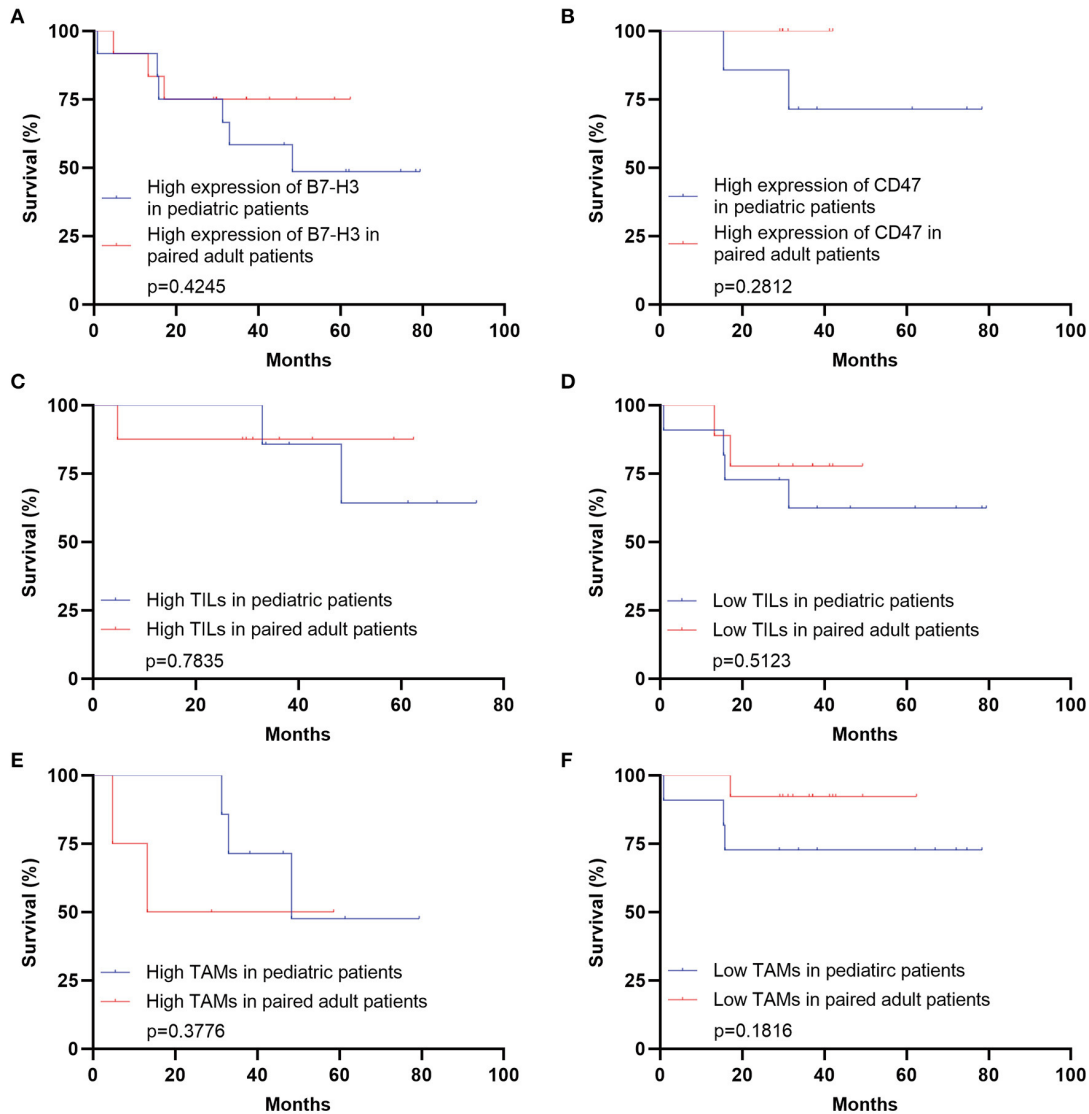
Survival analyses comparing pediatric and paired adult patients with DGs under a similar expression level of immune checkpoint molecules or TILs/TAMs. **(A)** Survival analysis comparing the pediatric group with the paired adult group under the high expression of B7-H3 of DGs (*p* = 0.4245). **(B)** Survival analysis comparing the pediatric group with the paired adult group under the high expression of CD47 of DGs (*p* = 0.2812). **(C)** Survival analysis comparing the pediatric group with the paired adult group under the high TILs of DGs (*p* = 0.7835). **(D)** Survival analysis comparing the pediatric group with the paired adult group under the low TILs of DGs (*p* = 0.5123). **(E)** Survival analysis comparing the pediatric group with the paired adult group under the high TAMs of DGs (*p* = 0.3776). **(F)** Survival analysis comparing the pediatric group with the paired adult group under the low TAMs of DGs (*p* = 0.1816). TILs refer to the tumor-infiltrating lymphocytes; TAMs refer to the tumor-associated macrophages.

The clinical immunotherapy strategies largely depend on the expression status of immune checkpoint molecules, which leads to different prognosis. We first performed the survival analysis comparing the 20 pediatric patients and 641 adult patients with DGs. Expectedly, the OS of adult patients was statistically shorter than the pediatric ones (Figure 4A). The result comparing 20 pediatric and 20 paired adult patients was not significant (Figure 4B). No significance was found analyzing the prognosis in pediatric patients according to the expression level of B7-H3, CD47, or TILs/TAMs, though an unfavorable trend was observed in the group with high expression of B7-H3 compared to the one with low expression (Figures 4C–F). For paired adult patients, the expression level of B7-H3 and CD47 had no statistical impact on OS (Figures 5A,B). Though the OS was not significantly affected by the expression of TILs, high TAMs remarkably shortened the survival time (Figures 5C,D). Moreover, the survival difference between the pediatric and paired adult patients under a similar expression level of checkpoints or TILs/TAMs was analyzed. However, no significance was found regarding the OS difference between the two groups (Figures 6A–F).

## Discussion

This is the first study analyzing the relationship between the expression of immune checkpoint molecules and the intracranial locations of pDGs after comparing to the adult counterparts.

DGs commonly affected adults, especially the middleaged and elderly patients. The WHO grade I gliomas were not included in the DGs, therefore the number of pediatric patients with DGs was limited and much lower than the adult counterparts. Glioblastoma, the most aggressive DGs, accounts for 53.9% in the cohort of 661 patients but only 15% in the pediatric cohort. We considered the difference of DGs incidence between pediatric and adult patients might be related to the intracranial location difference. By applying the ADIFFI method proposed by Ellingson et al., we found the left frontal lobe and right cerebellum were the preferred locations for pDGs. It was described that the cerebellum was the most vulnerable site affected by pilocytic astrocytoma (36.22%), which was the most common pediatric CNS tumors (22). The cerebellum might be a distinctive area to develop pilocytic astrocytoma and DGs in pediatric patients compared to the adults. The cerebellar location of pediatric high-grade glioma was reported to have a worse survival (23). A differential diagnosis for pilocytic astrocytoma and DGs in the pediatric cerebellum is necessary. Additionally, the anterior horn of ventricles is a significant location for adult DGs, which could be explained by the glioma origin from the subventricular zone (SVZ) (24).

Three immune checkpoints were typically selected to perform IHC staining in pediatric patients and paired adult patients who were chosen according to gender and histological types. B7-H3 (B7 homolog 3 protein), also known as CD276, belongs to the B7 superfamily (25). The roles of co-stimulator and co-inhibitor of B7-H3 during T-cell activation were reported, and the inhibition of B7-H3 checkpoint suppressed tumor growth by enhancing cytotoxic lymphocyte function (26). B7-H3 was highly expressed in gliomas and meningiomas, which could be treated by B7-H3-targeted CAR-T (27, 28). CD47 is overexpressed in hematologic and solid tumors, presenting the “don't eat me” signal against phagocytosis of macrophages after binding and activating signal regulatory protein–α (SIRPα) (29). It was demonstrated disrupting the CD47-SIRPα axis could exert antitumor effects on gliomas, and malignant pediatric brain tumors (30, 31). PD-L1 is the cognate ligand for PD-1, which is upregulated on tumor cells, and targeting the PD-1-PD-L1 axis is the robust immunotherapy (32). The expression of PD-L1 could be observed in GBM cells, which was the negative indicator for GBM outcome (33). The overexpression of B7-H3 and CD47 was validated in our results. However, PD-L1 was rarely detected. The difference in expression level between pediatric and adult DGs showed no statistical significance, indicating the similar expression patterns of immune checkpoints. The correlation analysis showed a potential association between the expression of PD-L1 and TAMs in pDGs, despite the low expression of PD-L1. It was described PD-L1 was expressed on TAMs in esophageal cancer and gastric cancer (34, 35). The positive relationship was also elucidated in pediatric cancers including Burkitt lymphoma, glioblastoma, and neuroblastoma (36). Therefore, in addition to B7-H3 and CD47, PD-L1 still remains to be the therapeutic target in pDGs. Interestingly, the activation of PD-L1^+^ NK cells with anti-PD-L1 inhibitor was the reason why some patients lacking PD-L1 expression on tumor cells still respond to anti-PD-L1 therapy (37).

Most importantly, the checkpoint-associated locations of DGs were found. Our results suggested the right posterior external capsule and the lateral side of the anterior horn of the left ventricle were predominant locations for the adult patients with high expression of B7-H3 and low expression of PD-L1 compared to pediatric ones, respectively. The external capsule is anatomically located between the putamen and claustrum, and is composed of white matter fibers. The results that DGs with high expression of B7-H3 in adults located in this region might exactly reveal the difference in age and the association between B7-H3 and DGs. As the white matter is indicated to contribute to the malignant behaviors such as the spread of gliomas (38), mature white matter in adults rather than pediatric patients is assumed to be more conducive to this cancerous nature. Moreover, the anterior horn (also known as the frontal horn) of the ventricle is found to frequently affected by adult DGs (Figure 1B). However, the ADIFFI indicated, compared with pediatric patients, adult DGs with low expression of PD-L1 in this region were statistically significant. The results were supposed to predict the efficacy of immunotherapies targeting PD-L1 for adult DGs in this risky area. Therefore, the findings might be valuable for the design of immunotherapy strategies and clinical trials, as exemplified by the fact that local immunotherapies such as the local CAR-T delivery and local radiotherapy acting as immunosensitizer might benefit from these statistically significant sites for therapeutic priority. However, the detailed biological mechanisms warrant further investigation, especially the laterality.

The survival analysis comparing the pediatric and paired adult patients under a similar expression level of checkpoints or TILs/TAMs showed no significance. But high expression of B7-H3 lead to a decreased survival in pDGs though no statistical significance was found (Figure 4C). A previous study investigated 47 pediatric glioma patients and found a significant relation between high expression of B7-H3 and poor prognosis (39). More importantly, Haydar and his colleagues demonstrated that B7-H3 was consistently expressed in pediatric brain tumors and the subsequent use of B7-H3-CAR-T cells resulted in remarkable tumor regression in patient-derived orthotopic xenografts (40). These findings collectively showed the B7-H3 could be a promising candidate of immunotherapy in pediatric gliomas.

There were several limitations in the current study. Firstly, the sample size of pDGs was limited. This is probably due to the objectively low incidence of pediatric diffuse gliomas compared with pilocytic astrocytoma, followed by the fact that substantial pediatric patients receive diagnosis and therapy in the local tertiary children's hospital. Further cooperation among medical centers to expand the sample size is necessary. According to the previous work by Ellingson et al. the method of correction for cluster-size using random permutations was conducted to remove the scattered clusters after ADIFFI (17, 18). However, the correction was not performed in the present work, as the ADIFFI results indicated concentrated areas with statistical significance, which were not scattered (Figure 3). Furthermore, the sample size of this study was relatively small. Thus, the statistic correction may be too conservative to find possible clusters of specific immune markers' expression. Additionally, other quantitative methods such as flow cytometry, and novel technologies such as sing-cell sequencing and mass cytometry would help researchers better understand the immune difference between pediatric and adult DGs. Furthermore, the mechanisms of immune checkpoint-associated locations of DGs warrant further investigation as mentioned.

In conclusion, our study indicated that in the context of spatial location difference between pediatric and adult DGs, though the expression level and the prognostic role of immune checkpoint molecules and TILs/TAMs were not significantly different, the immune checkpoint-associated locations of DGs were found, which might be valuable for the design of immunotherapy strategies and clinical trials.

## Data Availability Statement

The original contributions presented in the study are included in the article/supplementary material, further inquiries can be directed to the corresponding author/s.

## Ethics Statement

The studies involving human participants were reviewed and approved by the ethical committee on clinical human research of the Second Affiliated Hospital of Zhejiang University, School of Medicine. Written informed consent to participate in this study was provided by the participants' legal guardian/next of kin.

## Author Contributions

JZ and CS contributed to the study design. LZ, BZ, and JW collected and analyzed the imaging data. BZ and ZD performed the IHC staining. YI performed survival and statistical analyses. LZ, BZ, and ZD wrote the manuscript. YI, BJ, CS, and JZ reviewed all the data, results, and manuscript. All authors read and approved the final version of the manuscript.

## Conflict of Interest

The authors declare that the research was conducted in the absence of any commercial or financial relationships that could be construed as a potential conflict of interest.
